# Occult metastasis of hormone receptor-positive breast cancer to the ovary: A case report and literature review

**DOI:** 10.1097/MD.0000000000047830

**Published:** 2026-02-28

**Authors:** Pingping Li, Shaofeng Wu, Hailong Ma, Wenxin Ji, Yueyue Wang, Li Wang, Yinhai Dai

**Affiliations:** a Shaanxi University of Chinese Medicine, Xianyang, Shaanxi, China; b Department of Oncology Surgery, The Second Affiliated Hospital of Shaanxi University of Chinese Medicine, Xianyang, Shaanxi, China; c Department of Pathology, The Second Affiliated Hospital of Shaanxi University of Chinese Medicine, Xianyang, Shaanxi, China; d College of Basic Medicine, Shaanxi University of Chinese Medicine, Xianyang, Shaanxi, China.

**Keywords:** breast cancer, HR+/HER2−, metastatic carcinoma, neoadjuvant chemotherapy, oophorectomy

## Abstract

**Rationale:**

Breast cancer is the most common malignancy among women worldwide. It typically metastasizes to the bone, lungs, and liver, while ovarian involvement is relatively uncommon. This report aims to illustrate the clinical features, diagnostic approach, and treatment strategies for this rare type of metastasis through a case study, thereby enhancing clinicians’ awareness and management capabilities.

**Patient concerns:**

A 32-year-old premenopausal woman presented with a palpable nodule in the left breast. Comprehensive diagnostic evaluation, including mammography, ultrasonography, contrast-enhanced computed tomography, and core needle biopsy, confirmed invasive ductal carcinoma, classified as Luminal A subtype (estrogen receptor/progesterone receptor-positive, human epidermal growth factor receptor 2-negative).

**Diagnoses:**

Invasive ductal carcinoma of the left breast (pT3N3M1, stage IV) with ovarian metastasis.

**Interventions:**

The patient received 6 cycles of docetaxel/doxorubicin/cyclophosphamide chemotherapy (docetaxel, doxorubicin, and cyclophosphamide), followed by left modified radical mastectomy with axillary lymph node dissection, achieving R0 resection. Laparoscopic bilateral adnexectomy was also performed for ovarian ablation. Final pathology confirmed metastatic breast carcinoma in the ovaries.

**Outcomes:**

The patient successfully achieved surgical tumor reduction, recovered well postoperatively, and showed no clinical evidence of disease progression.

**Lessons:**

This case highlights the distinct characteristics of ovarian metastases in HR+/HER2− breast cancer and their critical importance in differential diagnosis, particularly in distinguishing them from primary gynecologic tumors. For patients with a history of breast cancer, the presence of pelvic lesions should prompt consideration of metastatic potential to guide appropriate comprehensive treatment.

## 1. Introduction

Breast cancer is one of the most common malignancies among women worldwide. According to the 2024 Global Cancer Statistics Report, it accounts for 32% of all newly diagnosed female cancers, ranking first and posing a serious threat to women’s health.^[1]^ Advances in targeted therapy and immunotherapy have significantly reduced breast cancer mortality.^[2]^ However, distant metastasis remains closely linked to poor clinical outcomes.

Breast cancer typically spreads through the lymphatic system but may also extend locally along ducts or fascial planes. Hematogenous dissemination can occur directly or through lymphatic invasion into the bloodstream. The most common metastatic sites include the skeleton, lungs, and liver, whereas ovarian metastasis is less common, observed in only 13% to 47% of metastatic cases.^[3]^ This article reports the comprehensive management of a patient with breast cancer and ovarian metastasis treated at the Department of Oncology and Breast Surgery, The Second Affiliated Hospital of Shaanxi University of Chinese Medicine. The patient underwent neoadjuvant chemotherapy followed by modified radical mastectomy with level I/II axillary lymph node dissection, combined with laparoscopic bilateral oophorectomy and thorough pelvic peritoneal inspection. A detailed discussion is also included.

## 2. Case presentation

A 32-year-old woman was admitted on May 17, 2023, with a recent diagnosis of left breast malignancy. She had first noticed a left subareolar mass 2 years earlier but did not seek medical attention. The mass was initially nontender and without nipple discharge or retraction but was fixed to underlying tissue. The recent increase in the size of the mass prompted her to seek evaluation at our hospital.

On examination, both breasts were symmetrical without peau d’orange or skin dimpling. The left nipple was retracted, and a hard, poorly defined, fixed mass measuring approximately 5.0 × 6.5 × 8.0 cm was palpable in the left breast. A firm, fixed lymph node measuring about 7.0 × 1.0 cm was also detected in the left axilla. The right breast and axilla were unremarkable.

**Figure 1.**
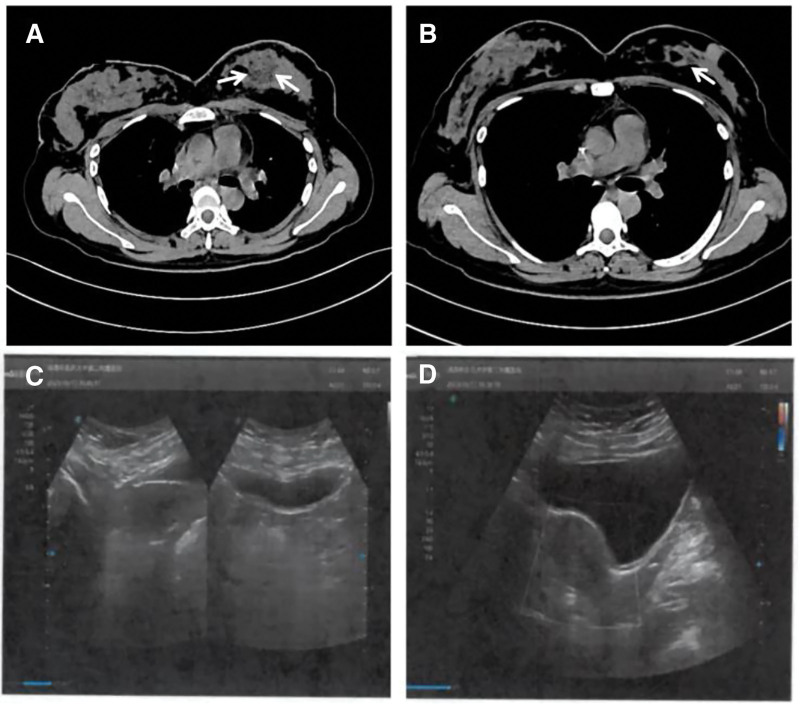
Prechemotherapy CT scan shows a circular hypodense lesion (A); postchemotherapy CT scan of the same slice (B) reveals marked reduction in the hypodense lesion, which is now difficult to discern. (C, D) Preoperative ultrasonographic images of the uterus and adnexa. No definite abnormal sonographic findings were observed in the uterus or bilateral adnexal regions. CT = computed tomography.

Breast ultrasonography performed on May 10, 2023, revealed a hypoechoic lesion in the left breast measuring approximately 47.0 × 66.8 × 78.2 mm (Fig. 1A). The lesion exhibited indistinct borders, spiculated margins, and a crab-like appearance infiltrating surrounding tissue, consistent with Breast Imaging Reporting and Data System Category 5. Enlarged lymph nodes were noted in the left axilla, the largest measuring 6.9 × 11.9 mm. Serum tumor markers were elevated, with carcinoembryonic antigen at 34.87 ng/mL and CA15-3 at 75.24 U/mL. A core needle biopsy on May 11, 2023, confirmed invasive carcinoma. Immunohistochemistry (IHC) revealed CK5/6 (−), human epidermal growth factor receptor (HER2) (0), Ki-67 (20%+), estrogen receptor (ER) (3+, 70%), progesterone receptor (PR) (2+, 20%), androgen receptor (3+, 90%), P53 (−), E-cadherin (+), and P120 (+).

Staging workup included upper abdominal computed tomography (CT), which revealed a ground-glass nodule in the right upper lung apex (recommended for follow-up), reduced transparency in both lungs suggesting small airway disease, bilateral pleural thickening and effusion, uneven thyroid density with a left breast soft tissue lesion requiring biopsy, no abnormalities in the brain, liver, gallbladder, pancreas, or spleen, and an old fracture of the right fifth axillary rib.

Based on biopsy results, laboratory findings, tumor size, and overall clinical status, neoadjuvant chemotherapy followed by surgery was recommended. The patient completed 6 cycles of the docetaxel/doxorubicin/cyclophosphamide regimen (docetaxel 75 mg/m^2^, doxorubicin 50 mg/m^2^, cyclophosphamide 500 mg/m^2^, every 21 days). After chemotherapy, the CA15-3 level decreased to 33.62 U/mL. Chemotherapy-induced bone marrow suppression was managed with anemia correction, leukocyte stimulation, and supportive care. Preoperative assessments were then completed.

Follow-up ultrasonography on October 17, 2023, showed a reduction of the left breast mass to 10.4 × 20.2 × 26.2 mm, classified as Breast Imaging Reporting and Data System Category 6. No enlarged axillary lymph nodes were detected (Fig. 1B). Abdominal ultrasound was unremarkable for the uterus and adnexa (Fig. 1C and D). A chest CT scan on October 18, 2023, showed a persistent right apical ground-glass nodule, bilateral pulmonary interstitial changes with pleural thickening, regression of the left breast lesion, heterogeneous thyroid density, no abnormalities in the brain, liver, gallbladder, pancreas, or spleen, and old fractures of the right fourth and fifth ribs.

**Figure 2.**
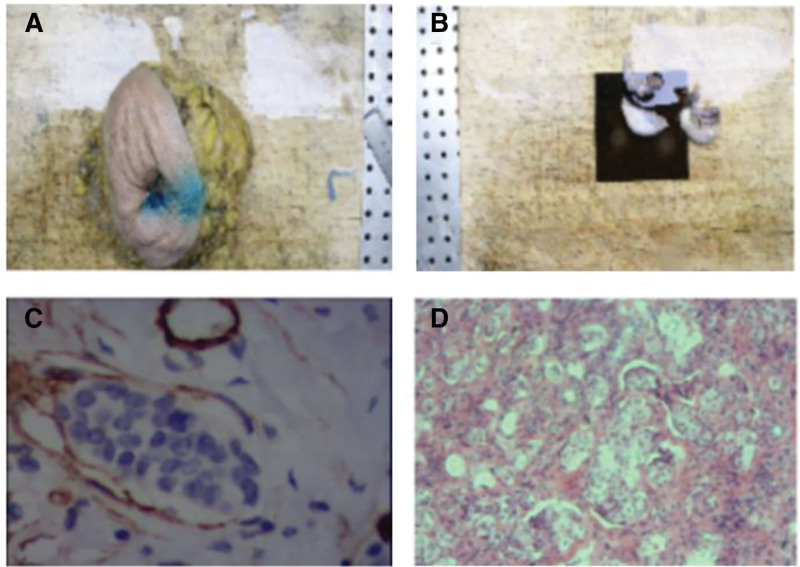
Pathological findings of the breast and ovarian lesions. (A, C) Gross macroscopic images of the affected breast after surgical resection, along with microscopic foci of cancer. (B, D) Gross macroscopic images of the ovaries following surgical resection, accompanied by microscopic cancer foci. Due to a hospital system update, some data were lost. This iconic image was extracted from the patient’s electronic report, and the clarity may not be optimal. A clearer pathological image can be found in Fig. 3.

**Figure 3.**
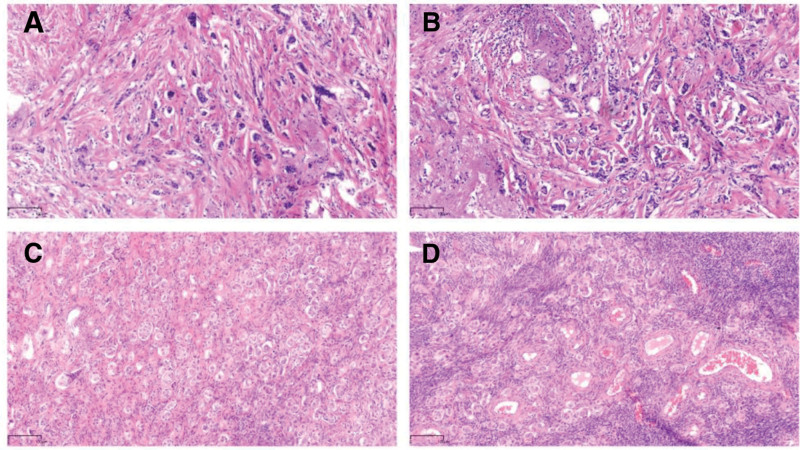
Histopathological features of invasive ductal carcinoma of the breast (H&E staining). (A) and (B) show irregular invasive nests and cords of tumor cells with moderate nuclear pleomorphism, infiltrating through a desmoplastic stroma. These features are consistent with an intermediate-grade (grade II) carcinoma. (C) and (D) show solid nests and clusters of malignant cells with moderate to severe nuclear pleomorphism, infiltrating the ovarian stroma. The morphological features are identical to the primary breast carcinoma, confirming metastatic breast cancer. H&E = hematoxylin and eosin.

On October 19, 2023, the patient underwent a left modified radical mastectomy. Postoperative pathology confirmed grade II invasive ductal carcinoma with vascular tumor emboli (Fig. 2A and C). Metastatic carcinoma was identified in 14 of 18 left axillary lymph nodes. No lobular features were identified. All surgical margins, including the nipple and base, were negative. IHC showed CK5/6 (−), HER2 (1+), Ki-67 (10%+), CD34 (−), ER (2+, 70%), PR (1+, 5%), androgen receptor (3+, 90%), P53 (−), E-cadherin (+), D2-40 (−), CD31 (−), and P120 (+) (Fig. 3A and B).

As a premenopausal woman with hormone receptor-positive disease, the patient was at increased risk of estrogen-driven progression. Considering her parity (2 children) and socioeconomic factors, bilateral salpingo-oophorectomy was recommended. After thorough counseling and informed consent, the procedure was performed laparoscopically on December 1, 2023. Preoperative adnexal ultrasound showed no abnormalities. Histopathological examination of the resected ovarian tissue confirmed metastatic breast carcinoma, consistent with the primary lesion based on IHC and clinical history (Fig. 2B and D). IHC of the ovarian specimen revealed HER2 (1+), Ki-67 (low index), ER (3+, 90%), PR (−), P53 (−), CA125 (+), PAX8 (−), P16 (+), vimentin (−), GATA-3 (+), and mammaglobin (−) (Fig. 3C and D).

Adjuvant endocrine therapy with letrozole and abemaciclib was initiated. The patient recovered well and was discharged with a final diagnosis of postoperative left breast cancer (pT3N3M1, stage IV). After excluding contraindications, adjuvant radiotherapy was initiated on January 8, 2024, in accordance with the guidelines of the Chinese Anti-Cancer Association to reduce recurrence risk. Treatment targeted the left chest wall and supraclavicular regions (clinical target volume: chest wall and supra-/infraclavicular nodes; planning target volume: clinical target volume + 0.5 cm), with a total dose of 50 Gy delivered in 25 fractions of 2 Gy, combined with deep hyperthermia. The patient experienced only mild radiation dermatitis and no other significant toxicities. Supportive care included continued endocrine therapy, nutritional support, management of cytopenias, and symptomatic treatment. Traditional Chinese medicine was also prescribed. The patient remained clinically stable during follow-up, with no major complications.

## 3. Discussion

Ovarian metastasis from breast cancer is uncommon but clinically significant. Diagnosis is often delayed because of nonspecific manifestations and the limited sensitivity of imaging examination, and many cases are discovered incidentally during autopsy or oophorectomy.^[3]^ This report describes the clinical course and molecular findings of a young premenopausal patient and discusses the pathogenesis, clinicopathological features, diagnostic challenges, and therapeutic strategies.

### 3.1. Pathogenesis

Although the mechanisms of ovarian metastasis from breast cancer are not fully understood, several pathways have been proposed. The ovaries’ abundant vasculature and lymphatic network create a favorable microenvironment for metastatic spread, with tumor cells potentially reaching the ovaries through hematogenous or lymphatic dissemination. In premenopausal women, the ovaries are the primary source of estrogen, and elevated hormone levels may promote the growth and metastatic potential of ERα-positive tumor cells. Germline *BRCA1/2* mutations also predispose patients to synchronous or metachronous involvement of the breast and ovaries. In addition, circulating tumor cells may reach the ovaries through the bloodstream and, supported by the local microenvironment, establish metastatic lesions, underscoring the complexity of organ-specific metastasis.^[4]^

### 3.2. Clinicopathological features

Ovarian metastases from breast cancer exhibit distinct clinicopathological characteristics. They occur predominantly in premenopausal women, are usually hormone receptor-positive, and most often originate from invasive ductal carcinoma. Clinical presentation is often subtle, with only a minority of patients experiencing symptoms such as vaginal bleeding, ascites, or pelvic pain. Although lobular carcinoma has a greater tendency for ovarian involvement due to its diffuse growth pattern and peritoneal involvement, our case illustrates that ovarian metastasis can also arise from pure invasive ductal carcinoma. In this patient, neither the primary breast tumor nor the axillary lymph node metastases exhibited lobular components. This observation highlights that ovarian metastasis should not be excluded solely based on histological subtype.

Given the nonspecific clinical features and limited sensitivity of imaging modalities, early diagnosis remains a significant challenge in clinical practice.

### 3.3. Differential diagnosis

When adnexal or ovarian masses are identified, clinicians often initially suspect a primary gynecologic or gastrointestinal malignancy rather than breast cancer metastasis. However, studies have shown that metastatic tumors account for approximately 5.2% to 10% of all ovarian neoplasms,^[5,6]^ with 57% arising from the gastrointestinal tract and 30% from breast cancer.^[5]^ These data highlight the importance of considering ovarian metastasis in patients with a history of breast cancer.

Distinguishing between primary ovarian tumors and metastases is essential, as treatment strategies differ substantially.^[7]^ Primary ovarian cancers are typically unilateral, are larger in size, and exhibit distinct histological features, whereas ovarian metastases are often bilateral, smaller, solid, and highly vascular. These characteristics may assist in preoperative ultrasonographic evaluation.^[8]^ The guiding vessel sign, defined as a dendritic vascular structure extending from the periphery toward the center of the lesion, has been suggested as a specific indicator of metastatic disease.^[9]^ Histopathological examination provides additional diagnostic clues, as metastatic deposits usually appear as isolated or multiple nodules embedded in otherwise normal ovarian stroma.^[10]^ By contrast, primary ovarian cancers often arise from the surface epithelium or superficial cortex and may be associated with occult fallopian tube carcinoma.^[11]^ IHC is also critical: GCDFP-15, mammaglobin, and GATA3 support a breast origin, whereas WT1, CA125, and PAX8 are more consistent with primary ovarian tumors.^[11–15]^

In the present case, preoperative imaging failed to reveal ovarian abnormalities, and the metastatic lesion was confirmed only on postoperative pathological examination. This finding underscores the limitations of imaging modalities such as ultrasound, magnetic resonance imaging, and CT in detecting early ovarian metastases or distinguishing them from primary ovarian tumors. Therefore, surgical exploration remains the gold standard for definitive diagnosis.

### 3.4. Treatment

Surgical resection remains the standard treatment for ovarian metastases from breast cancer. A study by Eitan et al involving 59 patients with pelvic metastatic breast cancer demonstrated improved survival in those who underwent optimal debulking surgery.^[16]^ Given that most patients with ovarian metastases are premenopausal and hormone receptor-positive, bilateral oophorectomy is recommended whenever feasible.^[17]^ Laparoscopy offers a minimally invasive approach for both biopsy and treatment, avoiding unnecessary laparotomy in benign conditions.^[18]^ In this patient, surgical castration not only achieved metastatic resection but also reduced endocrine stimulation and alleviated financial burden. Laparoscopic bilateral oophorectomy is associated with advantages such as minimal invasiveness, reduced blood loss, less postoperative pain, and faster recovery.^[16]^

### 3.5. Genetic profiling

To further characterize the molecular features of this case and explore the potential mechanisms of ovarian metastasis, targeted next-generation sequencing using an Illumina platform (Illumina, Inc., San Diego) was performed on both the primary breast tumor and the ovarian metastasis (Tables 1 and 2). The analysis revealed *PIK3CA* p.E542K and *FGFR3* p.I538V mutations in the primary tumor, which were retained in the ovarian metastasis. In addition, the metastasis acquired a novel *PIK3CA* p.M1004I mutation, suggesting further clonal evolution during the metastatic process. No pathogenic variants were identified in *BRCA1/2*, and the tumor demonstrated microsatellite stability.

**Table 1:** Genetic profiling results of the primary breast carcinoma.

Name	Mutation status	Mutation site
*AKT1*	Unmutated	–
*EPCAM*	Unmutated	–
*FGFR3*	Mutated	p.I538V
*NTRK2*	Unmutated	–
*RET*	Unmutated	–
*BRAF*	Unmutated	–
*ERBB2*	Unmutated	–
*MLH1*	Unmutated	–
*NTRK3*	Unmutated	–
*TP53*	Unmutated	–
*BRCA1*	Unmutated	–
*ESR1*	Unmutated	–
*MSH2*	Unmutated	–
*PALB2*	Unmutated	–
*BRCA2*	Unmutated	–
*FBXW7*	Unmutated	–
*MSH6*	Unmutated	–
*PIK3CA*	Mutated	p.E542K
*CCND1*	Unmutated	–
*FGFR1*	Unmutated	–
*MTOR*	Unmutated	–
*PMS2*	Unmutated	–
*EGFR*	Unmutated	–
*FGFR2*	Unmutated	–
*NTRK1*	Unmutated	–
*PTEN*	Unmutated	–

The table lists the tested genes and their corresponding mutation status and specific mutation sites.

**Table 2:** Genetic profiling results of the metastatic carcinoma to the ovary.

Name	Mutation status	Mutation site
*AKT1*	Unmutated	–
*EPCAM*	Unmutated	–
*FGFR3*	Mutated	p.I538V
*NTRK2*	Unmutated	–
*RET*	Unmutated	–
*BRAF*	Unmutated	–
*ERBB2*	Unmutated	–
*MLH1*	Unmutated	–
*NTRK3*	Unmutated	–
*TP53*	Unmutated	–
*BRCA1*	Unmutated	–
*ESR1*	Unmutated	–
*MSH2*	Unmutated	–
*PALB2*	Unmutated	–
*BRCA2*	Unmutated	–
*FBXW7*	Unmutated	–
*MSH6*	Unmutated	–
*PIK3CA*	Mutated	p.E542K, P.M1004I
*CCND1*	Unmutated	–
*FGFR1*	Unmutated	–
*MTOR*	Unmutated	–
*PMS2*	Unmutated	–
*EGFR*	Unmutated	–
*FGFR2*	Unmutated	–
*NTRK1*	Unmutated	–
*PTEN*	Unmutated	–

The table lists the tested genes and their corresponding mutation status and specific mutation sites. Note the additional mutation in *PIK3CA* (p.M1004I) compared to the primary breast tumor.

Molecular subtyping indicated that this case belonged to the HR+/HER2− subtype, which accounts for approximately 60% of metastatic breast cancers.^[19]^ Genomic studies have shown that *PIK3CA* is one of the most frequently mutated genes in HR+/HER2− breast cancer and is commonly associated with poor prognosis.^[20]^ Such mutations result in constitutive activation of the PI3K/AKT/mammalian target of rapamycin pathway, thereby promoting cell proliferation, metabolism, survival, and cytoskeletal remodeling, ultimately driving tumor progression.^[21]^ At the same time, *FGFR3* encodes a receptor tyrosine kinase that plays a pivotal role in cell proliferation, migration, and angiogenesis, and acts synergistically with vascular endothelial growth factor signaling to promote tumor neovascularization.^[22]^

*BRCA1/2* mutations are closely associated with hereditary breast and ovarian cancers.^[23]^ In this case, the absence of *BRCA1/2* mutations indicates that the patient was not predisposed to hereditary cancer, making it more likely that the ovarian lesion represented metastasis rather than a new primary tumor. The concordant *PIK3CA* and *FGFR3* mutational profiles identified in both the primary and metastatic tumors provide strong molecular evidence for a metastatic origin and also suggest the emergence of additional genetic events during clonal evolution. Together, these findings deepen our understanding of the molecular mechanisms underlying ovarian metastasis from breast cancer and provide important implications for individualized therapy, targeted drug development, and future mechanistic research.

## 4. Conclusions

Ovarian metastasis from breast cancer is uncommon and often remains undiagnosed due to its nonspecific symptoms and the limited sensitivity of imaging techniques, which frequently lead to delayed diagnosis. In this case, the ovarian lesion was not detected preoperatively and was only identified postoperatively through pathological examination, emphasizing the diagnostic challenge posed by occult metastasis.

Clinically, this case underscores that ovarian metastasis can occur even in pure invasive ductal carcinoma, not solely in lobular carcinoma. As such, clinicians should maintain a high index of suspicion for ovarian metastasis in patients with a history of breast cancer, even in the absence of imaging abnormalities. It is crucial to avoid attributing adnexal masses solely to primary gynecologic tumors and to consider metastatic disease as a differential diagnosis. Timely surgical exploration, when appropriate, can provide a definitive diagnosis and improve patient outcomes.

From a research perspective, molecular profiling of the primary and metastatic lesions revealed concordant mutations, while also suggesting the presence of additional genetic events that emerged during the metastatic process. These findings enhance our understanding of the molecular mechanisms underlying ovarian metastasis from breast cancer and highlight the importance of molecular testing in such cases. Future studies should focus on the genetic and molecular drivers of ovarian involvement in breast cancer metastasis, with the goal of enabling earlier detection and developing more effective individualized treatment strategies.

Furthermore, this case provides a paradigm of occult ovarian metastasis in HR+/HER2− breast cancer, illustrating how a combination of clinical suspicion, surgical intervention, pathological scrutiny, and molecular profiling can lead to accurate diagnosis and effective management. It reinforces the need for heightened awareness of nonclassical metastatic patterns and advocates for individualized, multidisciplinary strategies in advanced breast cancer care.

Finally, this study has several limitations inherent to its design as a single-center retrospective case report. First, the findings from a single patient cannot be generalized to the broader population of breast cancer patients. The retrospective nature may introduce selection bias and data incompleteness. The occult ovarian metastasis was undetectable by preoperative imaging, highlighting the limited sensitivity of current modalities for early detection. Furthermore, while targeted next-generation sequencing provided insights, the selected gene panel may not capture all relevant alterations, and the functional implications of the identified mutations remain unvalidated. These limitations underscore the need for larger prospective studies to validate the observations and optimize management strategies.

## Author contributions

**Conceptualization:** Hailong Ma.

**Funding acquisition:** Yinhai Dai.

**Methodology:** Shaofeng Wu.

**Investigation:** Shaofeng Wu, Wenxin Ji.

**Validation:** Yueyue Wang.

**Project administration:** Yinhai Dai.

**Writing – original draft:** Pingping Li.

**Writing – review & editing:** Li Wang, Pingping Li.
